# Generic health literacy measurement instruments for children and adolescents: a systematic review of the literature

**DOI:** 10.1186/s12889-018-5054-0

**Published:** 2018-01-22

**Authors:** Orkan Okan, Ester Lopes, Torsten Michael Bollweg, Janine Bröder, Melanie Messer, Dirk Bruland, Emma Bond, Graça S. Carvalho, Kristine Sørensen, Luis Saboga-Nunes, Diane Levin-Zamir, Diana Sahrai, Uwe H. Bittlingmayer, Jürgen M. Pelikan, Malcolm Thomas, Ullrich Bauer, Paulo Pinheiro

**Affiliations:** 1 Faculty of Educational Science, Centre for Prevention and Intervention in Childhood and Adolescents (CPI), Bielefeld University, Bielefeld, NRW Germany; 2 School of Public Health, Public Health Nursing & Health Science Research, Bielefeld University, Bielefeld, NRW Germany; 3 University of Suffolk, Ipswich, England UK; 4 CIEC, Institute of Education, University of Minho, Braga, Portugal; 5 Global Health Literacy Academy, Urmond, The Netherlands; 6 CIESP, National School of Public Health, ISAMB (FMUL), Universidade NOVA de Lisboa, Lisbon, Portugal; 7 School of Public Health, University of Haifa, Haifa, Israel; 8 School of Education, University of Applied Sciences and Arts, Northwestern Switzerland, Basel, Switzerland; 9 University of Education, Freiburg, i.Br Germany; 10 Austria & Institute for Public Health, University of Vienna, Vienna, Austria; 11 School of Education, Aberystwyth University, Aberystwyth, Wales; 12 Clalit Health Services, Department of Health Education and Promotion, Tel Aviv, Israel

**Keywords:** Health literacy, Measurement, Assessment, Instrument, Children, Adolescents, Literature review

## Abstract

**Background:**

Health literacy is an important health promotion concern and recently children and adolescents have been the focus of increased academic attention. To assess the health literacy of this population, researchers have been focussing on developing instruments to measure their health literacy. Compared to the wider availability of instruments for adults, only a few tools are known for younger age groups. The objective of this study is to systematically review the field of generic child and adolescent health literacy measurement instruments that are currently available.

**Method:**

A systematic literature search was undertaken in five databases (PubMed, CINAHL, PsycNET, ERIC, and FIS) on articles published between January 1990 and July 2015, addressing children and adolescents ≤18 years old. Eligible articles were analysed, data was extracted, and synthesised according to review objectives.

**Results:**

Fifteen generic health literacy measurement instruments for children and adolescents were identified. All, except two, are self-administered instruments. Seven are objective measures (performance-based tests), seven are subjective measures (self-reporting), and one uses a mixed-method measurement. Most instruments applied a broad and multidimensional understanding of health literacy. The instruments were developed in eight different countries, with most tools originating in the United States (*n* = 6). Among the instruments, 31 different components related to health literacy were identified. Accordingly, the studies exhibit a variety of implicit or explicit conceptual and operational definitions, and most instruments have been used in schools and other educational contexts. While the youngest age group studied was 7-year-old children within a parent-child study, there is only one instrument specifically designed for primary school children and none for early years.

**Conclusions:**

Despite the reported paucity of health literacy research involving children and adolescents, an unexpected number of health literacy measurement studies in children’s populations was found. Most instruments tend to measure their own specific understanding of health literacy and not all provide sufficient conceptual information. To advance health literacy instruments, a much more standardised approach is necessary including improved reporting on the development and validation processes. Further research is required to improve health literacy instruments for children and adolescents and to provide knowledge to inform effective interventions.

**Electronic supplementary material:**

The online version of this article (10.1186/s12889-018-5054-0) contains supplementary material, which is available to authorized users.

## Background

Health literacy is currently experiencing increased attention in contemporary research, practice, and policy [1–7]. In health promotion, health literacy is understood to be an empowering resource for individuals, related to education and linked to literacy. It comprises the skills, knowledge, and motivation to access, understand, and appraise health-related information in order to apply informed health decisions in everyday life [1]. Although health literacy remains content specific, contextual factors impact on the practice of health literacy as well, and health literacy should ideally improve individual health behaviour [8]. In addition, health literacy is a double-sided concept that encompasses individual capabilities as well as system demands and complexities, which influence health behaviours and health-related interactions [9, 10].

Current research links limited health literacy to a lack of health knowledge, poor disease management skills, medication treatment errors, inadequate health communication skills, difficulties in navigating the healthcare system, poor access to healthcare services, increased healthcare costs, and poorer health outcomes [11]. In Europe, the European Health Literacy study (HLS-EU) conducted in eight countries found that an average of 47% of all respondents had limited health literacy [10]. Most of these studies were conducted among adults, and comparatively, the scientific literature on child and adolescent health literacy lacks evidence. Although health literacy is rooted in school health education aimed at improving children’s health literacy [12], children and adolescents have been given little attention in health literacy studies in past decades [13–16]. This contradicts with the importance given to childhood and adolescence for the development of health skills, health-related knowledge, and healthy behaviours and practices [17–19].

Many scholars argue, in accordance with findings from developmental research, that effective health literacy development begins in early childhood [13, 20, 21] and that schools are viewed as major settings for early health literacy promotion [22–24]. Recently, there have been some remarkable efforts towards performing research with younger age groups [14, 25–30]. Interestingly, due to the growing attention paid to children and adolescents for health literacy development, health literacy promotion in early childhood has been exclusively included in a policy brief of the World Health Organization (WHO) on investing in health literacy in the European Region [6] and in their recently published Shanghai declaration on health promotion [7].

To identify the needs of children and adolescents and to address specific target areas for action, validated and reliable measurement instruments to assess health literacy are crucial [5, 26, 27]. Although over a hundred instruments measuring either specific or generic health literacy in adults have been identified in several systematic and/or scoping reviews [4, 31–37], to date there is only one systematic review on child and adolescent measurement tools [38]. This review identified 16 tools comprising both generic and specific health literacy instruments developed between 2007 and 2011 with mixed results suggesting that available tools are not adequately measuring and depicting health literacy. Furthermore, the authors of that review suggest that future research regarding concepts and measurements should shift away from a healthcare perspective to a health promotion and education perspective instead. Another potential criticism arises from the fact that each instrument uses its own specific understanding of health literacy, which makes it difficult to compare results across studies.

This present systematic review, therefore, aims to identify, retrieve, analyse, and assess available generic health literacy measurement instruments for children and adolescents ≤18 years old. To specifically and exclusively focus on generic health literacy tools only, measurement tools for domain-specific health literacies, such as mental health literacy, oral health literacy, eHealth literacy, and media health literacy, were excluded from this study. In this review specific attention will be paid to the following:

- instrument characteristics;
- country of origin and setting;
- target or age group;
- questionnaire administration mode;
- participant participation in the development process of the questionnaires;
- psychometric properties;
- contextual factors;
- underlying health literacy models/definitions; and
- scope of measured components.

This systematic literature review was conducted as part of the MoMChild project (Methods of Measuring Health Literacy in Children), which is part of the German Health Literacy in Childhood and Adolescence Research Consortium (HLCA).

## Method

**Fig. 1:**
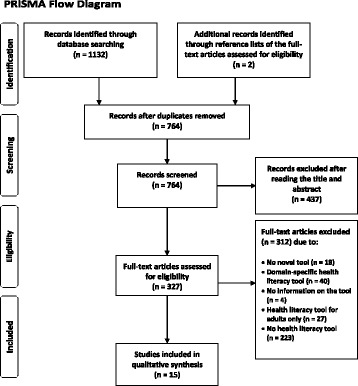
PRISMA Flow Diagram

The research team conducted a systematic review of the literature on health literacy measurement instruments for children and adolescents ≤18 years old. To ensure transparency and completeness of the research and to improve the reporting of this review, the 27-item checklist of the Preferred Reporting Items for Systematic reviews and Meta-Analyses (PRISMA statement) was adopted and included in the supplementary information files of this article. The PRISMA flow diagram (see Fig. 1) illustrates the inclusion and exclusion process [39].

### Data sources, search strategy, and study selection

Three researchers (OO, MM, PP) developed the search strategy/method and algorithm. Two researchers (OO, EL) independently searched the databases using identical search algorithms for the respective databases and analysed the data. Three researchers (TMB, MM, JB) checked the initial results, and five researchers (OO, TMB, MM, JB, PP) critically discussed the search outcomes.

### Data sources

The search was performed in five databases: Medline via PubMed, the Cumulative Index to Nursing and Allied Health Literature (CINAHL) via EBSCOhost, the American Psychological Association (APA) PsycNET search platform, the Educational Resources Information Center (ERIC), and the German Fachinformationssystem Bildung (FIS).

### Search strategy

The five abovementioned databases were initially searched from May – July 2015 using a composite search term that comprised a combination of three terms for papers published between January 1990 and July 2015. To combine search terms Boolean operators (AND/OR) were used. Wildcard characters were used in order to cover all spelling variations of the search terms. The first search term was “health literacy”, the second addressed the target population (“child*”, “adolescen*”, or “youth”), and the third term focused on either one of ten common terms/topics in the context of measurement tools (“measur*”, “test*”, “tool*”, “instrument*”, “questionnaire*”, “assessment*”, “screen*”, “survey*”, “psychometric*”, and “review*”). In the FIS, the search terms “Gesundheitsbildung” and “Gesundheitskompetenz” were used. Additionally, reference lists of included full-text articles were searched manually (OO) for potentially relevant publications. The following algorithm has been used in PubMed, while the search strategy used in PubMed was adapted to the specifications of the other four databases (see Additional files 1 and 2).

((health literacy[Title/Abstract]) AND ((child*[Title/Abstract]) OR (adolescen*[Title/Abstract]) OR (youth[Title/Abstract])) AND ((measur*[Title/Abstract]) OR (test*[Title/Abstract]) OR (tool*[Title/Abstract]) OR (instrument*[Title/Abstract]) OR (questionnaire*[Title/Abstract]) OR (assessment*[Title/Abstract]) OR (screen*[Title/Abstract]) OR (survey*[Title/Abstract]) OR (psychometric*[Title/Abstract]) OR (review*[Title/Abstract])))

### Study selection

For the purposes of this search, the following inclusion criteria were used (see Table 1): (a) time of publication between January 1990 and July 2015 (as the first health literacy tool was introduced in the early 1990s); (b) English or German language; (c) original publication describing the first use of a health literacy instrument; (d) a study population of children and/or adolescents or at least including these; (e) any setting, i.e., healthcare, schools or other; and (f) any country.

**Table 1:** Inclusion and exclusion criteria

Criterion	Inclusion	Exclusion
Time	January 1990 – July 2015	Studies before 1990 and after July 2015
Language	English, German	Any other language
Type of publication	Original papers published in peer-reviewed journals, or reports	Any non-original publication, any editorials, letters to editors, theses, books
Focus of study	Any study reporting on a generic health literacy instrument, its first-time use, development or validation process	Any study reporting on a domain-specific health literacy instrument (i.e. mental health literacy, media or eHealth literacy), and any non-health literacy instrument
Study population	Articles including children and adolescents ≤18 years	Any population older > 18 years
Setting	Any setting	Nil
Country	Any country	Nil

### Screening, data extraction, and analysis

Five researchers (OO, EL, TMB, MM, JB) participated in the screening process. Screening took place in two steps: (1) title/abstract screening was performed by two researchers (OO, EL) independently, and (2) full-text analysis of the eligible publications was performed by three researchers (OO, EL, MM). Two researchers (TMB, JB) checked the results independently. To find consensus and validate the findings, expert consultations were performed by approaching the other authors (DB, EB, GSC, KS, LSN, DLZ, DS, UHB, JP, MT, UB, PP). Literature was imported to the reference management software CITAVI 5. The characteristics of the health literacy measurement instruments identified in the relevant publications were analysed and extracted by two researchers independently (OO, EL) based on the objectives of this review. The decision on what data to extract from the articles was based partly on (a) former systematic reviews of health literacy measurement tools [4, 38] and (b) an extensive discussion and consensus among the authors of this review and with further health literacy experts from the HLCA research consortium. If information on the validity and reliability of instruments was documented within the articles, these data were extracted and analysed as well.

## Results

This review focused exclusively on generic health literacy measures. The search process identified *N* = 1132 publications matching the search criteria (PubMed *n* = 291, CINHAL *n* = 201, PsycNET *n* = 357, ERIC *n* = 226, FIS *n* = 57). The manual search led to the identification of an additional n = 2 articles (see Fig. 1: PRISMA flow diagram). After removing duplicates, *n* = 764 articles remained, of which a further *n* = 437 articles were excluded after screening the titles and abstracts. A total of *n* = 327 articles underwent full-text analysis. Finally, all articles not matching the inclusion criteria (*n* = 312) were excluded from qualitative synthesis yielding *N* = 15 articles reporting 15 different questionnaires.

### Instrument characteristics

Among the identified instruments (see Table 2), ten instruments are novel instruments that were developed especially either for children and/or adolescents [40–47] or for adult age groups including adolescents 15 years and older [48, 49]. In relation to the teen version of the Rapid Estimate of Adolescent Literacy in Medicine (REALM-teen) [50], the adolescent version of the Test of Functional Health Literacy in Adults (TOFHLAd) [51], and the Newest Vital Sign (NVS) [52], our review identified child and adolescent adaptations of the most widely used fast-screening instruments in clinical and medical-related adult health literacy research. Another adaptation/validation study conducted in Austria [53] applied the population-based health literacy tool HLS-EU-Q47, which was developed for the HLS-EU [10]. This tool, which was originally developed and validated for adults (15 years +), was specifically validated as a long form of the tool and subsequently adapted as a short form for adolescents. Finally, one of the identified instruments is a shorter form of an already existing health literacy measurement tool for adolescent mothers, but analysis of the longer form of the tool is yet not published [54]. REALM-teen was the first health literacy instrument ever to be applied in a child population during a validation study [50]. The most recent study to use this instrument was conducted with the Maternal Health Literacy (MaHeLi) scale in Uganda in 2015. Questionnaire items are fully provided in seven of the studies [40, 43, 47, 49, 52–54] but are not provided at all in seven other studies [41, 42, 44, 45, 48, 50, 51]. One study provides only some of the items [46].

**Table 2:** Health literacy instruments for children and adolescents

No.	Author, year country language	Instrument	Type	Study aim	Sample size, study population, setting	Scope of measured components	Items, response format	Time of administration	Reliability	Validity, responsiveness and sensitivity
1	Davis et al., 2006 [50] USA English	Rapid Estimate of Adult Literacy in Medicine - Teen (REALM-teen) Adaptation of an existing instrument for adults (REALM)	Objective measurement/performance based assessment	Validation of the REALM-teen for adolescents	*N* = 1533 10–19 y. participants were 50% black, 53% female; 34% were enrolled in middle school and 66% in high school Secondary school Mixed setting, schools and healthcare setting	Word recognition; Pronunciation	66 items (not provided) Health words arranged in increasing order of difficulty Pronouncing words	Usually 2–3 min, here approx. 3 min	Internal consistency *α* = .94	Convergent: SORT-R *r* = .93 WRAT *r* = .83 Receiver operating characteristic (ROC) analysis: SORT-R Area under ROC (AUC) = .84
2	Brown et al., 2007 [40] USA English	KidsHealth KidsPoll of Health Literacy New instrument based on National Health Education Standards (NHES)	Subjective measurement/self-report	Investigating health literacy and its effects on health behaviour and practice	*N* = 1178 9–13 y. 5th – 8th grade Secondary school 11 health education centres	Ability to understand, access and apply health information; Interest in health; Belief: Ability that health behaviour is affective; Attitude	8 items (provided) Remote keypads Close-end questions with a maximum of 5 answer choices	Not available (n.a.)	n.a.	n.a.
3	Hubbard and Rainey 2007 [41] USA English	“Health Literacy Instrument” no name. New instrument based on Health Education Assessment Project (HEAP)	Objective measurement/performance based assessment (not explicitly stated by the article but indicated in the text)	To evaluate the influence of comprehensive textbook-based instruction on students’ acquisition of health-related concepts and skills	*N* = 669 secondary school children and adolescents *n* = 333 female; *n* = 333 male; *n* = 3 missing (Treatment group: *n* = 330; control group: *n* = 339) School setting, *N* = 3 schools (*n* = 2 middle; *n* = 1 high school)	Understanding of health concepts about tobacco, physical activity, and nutrition; Ability to access information, interpersonal communication, decision-making, goal setting, and self-management	30 items (not provided) 15 items related to health concepts 15 items related to demonstrate skills	n.a.	Reliability coefficient of the concepts scale α = .76 middle school; α = .72 high school; of the skills scale α = .75 middle school; α = .76 high school HEAP assessment items proved to be reliable	Not provided, HEAP assessment items proved to be valid
4	Chisolm and Buchanan, 2007 [51] USA English	Test of Functional Health Literacy in Adults (TOFHLA) in adolescent population (TOFHLAd), 2 components TOFHLA-R & TOFHLA-N Adaptation of an existing instrument for adults	Objective measurement/performance based assessment	Pilot validation for adolescents	*N* = 50 13–17 y. *n* = 26 female; *n* = 24 male Healthcare setting	Reading comprehension (TOFHLA-R); Numeracy (TOFHLA-N)	67 items (not provided), 2 components; TOFHLA-R has 50 reading comprehension items, cloze procedure; TOFHLA-N has 17 numeracy items	10–20 min: average of 12.9 min with a range from 8.9 to 17.3	n.a.	Concurrent: TOFHLA-R: WRAT3 *r* = .59 (*p* < .001) REALM *r* = .60 (p < .001) TOFHLA-N: WRAT3 *r* = .11 (*p* = .45) REALM *r* = .18 (*p* = .22)
5	Steckelberg et al., 2009 [48] Germany German	Critical Health Competence Test (CHC) New instrument	Objective measurement/performance based assessment	Development and validation of a questionnaire to measure critical health competencies	*N* = 429 15–42 y. *n* = 322 (first field test); *n* = 255 10th and 11th from secondary schools, *n* = 67 university students *n* = 107 (second field test); *n* = 94 secondary schools, *n* = 13 university students	A. Understanding medical concepts; B. Skills of searching literature (information seeking); C. Basic statistics (numeracy); D. Design of experiments and sampling	72 items (not provided); Scenarios (S) (items): S1 (16), S2 (20), S3 (15), S4 (21). A, 15 items B, 22 items C, 18 items D, 17 items	Should not exceed 90 min	Rasch analysis: Mean person parameter S1: 395 S2: 497 S3: 635 S4: 473	Construct validity: Cohen’s d = 4.33 [95% CI 3.51–5.16] Rasch model WINMIRA ANOVA = .91
6	Vardavas et al., 2009 [42] Greece Greek	Health Literacy Questionnaire for Children New instrument	Subjective measurement/self-report	To locate the topics and to assess the sources of health information of adolescents	*N* = 369, 12–18 y. 46.6% male; 53,4% female; 97, 3% Greek nationality Secondary school children from urban areas of Athens and Crete	Questions on health education topics; Access and source of health information (seeking); Stated satisfaction	n.a. no information on specific items is available	n.a.	n.a	n.a.
7	Schmidt et al., 2010 [43] Germany German	GeKoKids Questionnaire New instrument	Mixed approach	To elaborate a set of short scales to measure important health literacy domains in children; and To analyse their associations among each other	*N* = 852; aged 9–13 y., Germany *n* = 401 female *n* = 451 male *n* = 29 migration background Secondary school	Knowledge; Attitudes; Communication; Behaviour; Self-efficacy	17 items (provided) Knowledge: 3 items Communication: 3 items Attitude: 4 items Behaviour: 4 items Self-efficacy: 3 items	n.a.	Internal consistencies communication α = .73 attitude α = .57 Rasch analysis: Knowledge χ^2^ = 6.45, *P* = 0.17 Behaviour χ^2^ = 15.48, *P* = 0.12	n.a.
8	Wu et al., 2010 [44] Canada English	Health literacy instrument for high school students New instrument	Objective measurement/performance based assessment	Development and validation of a health literacy measurement tool for high school students in classrooms	*N* = 275 secondary school children 8th *n* = 2 9th *n* = 34 10th *n* = 202 11th *n* = 16 12th *n* = 17 48% male 52% female 69,1% other language than English at home 30,09% language English at home	Understand; Evaluate	47 items (not provided) open-ended following health related reading passages Understand: 30 items Evaluate: 17 items	n.a.	Internal consistency: α = .92	Convergent: Age, *r* = .17 Male gender, r = .18 Age came to Canada, r = .22 Non-English speaker, *r* = .15 Mother’s edu, *r* = .19 Father’s edu, *r* = .22 GPA, *r* = .48 Time reading/ study, *r* = .40 ‘fair’
9	Yu et al., 2012 [45] China Chinese	Health Literacy Questionnaire New instrument	Subjective measurement/self-report	To assess the students’ health literacy gained through school health education	*N* = 8008 Elementary School (*n* = 77) Pupils *n* = 4011 Middle School (*n* = 76) Pupils *n* = 3997	Knowledge; Attitude; Practice (health behaviour and lifestyle)	37 items (not provided) close-ended and open-ended	n.a.	Internal consistency: α = .0.73 Spearman-Brown coefficient 0.75	n.a.
10	Chinn et al., 2013 [49] England English	All Aspects of Health Literacy Scale (AAHLS) New instrument	Subjective measurement/self-report	To develop a health literacy instrument to use in primary care settings	*N* = 146 Range: 15–82 y. *n* = 114 female; *n* = 32 male Ethnicity Asian: 81 Black: 5 Mixed race: 2 White: 51 Other: 7 Healthcare setting	Functional HL; Communicative HL; Critical HL	14 items (provided) Functional: 4 items Communicative: 3 items Critical: 7 items	Approx. 7 mins.	Internal consistency: α = .75 Functional HL α = .82 Communicative HL α = .69 Critical HL α = .42	Convergent: Functional vs Communicative, *r* = .39 Functional vs Critical, *r* = .59 Communicative vs Critical, r = .19
11	Wallmann et al., 2012 [46] Germany German	Health Quiz New instrument	Objective measurement / performance based assessment	To measure and assess health knowledge as part of health literacy	*N* = 699 7th grade adolescents in Germany *N* = 375 male; *n* = 324 female 4 school types: Gymnasium; secondary school (*n* = 195), Realschule; secondary modern/intermediate school (*n* = 231), Gesamtschule; comprehensive school (*n* = 81), general school; Hauptschule (*n* = 192). School setting	Knowledge	49 items (partly provided) Nutrition: 7 items; Prevention: 6 items; Spare time activity (health promotion): 4 items; Human body: 32 items (12 Items health behaviour / 20 items human anatomy) Response: 4 response categories, only one right choice	20 min	n.a.	n.a.
12	Massey et al., 2013 [47] USA English	Multidimensional health literacy instrument New instrument	Subjective measurement / self-report	To develop a multidimensional health literacy instrument for adolescents concerning the health environment This study was part of a larger study that examined the effectiveness of a health literacy intervention	*N* = 1208 13–17 y. Mean age 14.8 y. Over 60% females Hispanic/Latino 33.7% White 22.1% Black 13.2% Asian 7.9% Other 1.9% Multi-Ethnic 20.4% Healthcare setting	(1) Patient-provider encounter; (2) Interacting with the healthcare system; (3) Rights and responsibilities; (4) Health information seeking; (5) Confidence in health information from personal source; (6) Confidence in health information from media source	24 items (provided) (1) 4 items (2) 5 items (3) 7 items (4) 3 items (5) 3 items (6) 3 items 5 point Likert scale paper and pencil or online	n.a.	Internal consistency: α = .834 Corresponding factors (related to six dimension) 1: α = .815 2: α = .803 3: α = .827 4: α = .638 5: α = .834 6: α = .709	n.a.
13	Röthlin et al., 2013 [53] Austria German	HLS-EU-Q47 and -Q16 Existing tool	Subjective measurement / self-report	To apply and validate the HLS-EU instrument in the Austrian youth population	*N* = 571 15 y. Female 52.7% Male 46.8% No answer 0.5% Parents born in Austria 84.1% One parent born in Austria 7% Not born in Austria 9% No setting information	Access; Understand; Appraise; Apply	47 items provided including short scale of 16 items Dichotomous response format (easy and difficult) Access: 4 items Understand: 6 items Appraise 3 items Apply: 3 items	n.a.	Internal consistency α = .90 Healthcare: α = .69 Disease prevention: α = .81 Health promotion: α = .81	Convergent: HLS-EU-Q4 and: NVS *r* = .09 HLS-EU-Q16 and NVS *r* = .14 Concurrent: HLS-EU-Q16 *r* = .82
14	Driessnack et al., 2014 [52] USA English	Newest Vital Sign Existing instrument	Objective measurement / performance based assessment	To explore the feasibility, utility, and validity of using the Newest Vital Sign (NVS) tool to assess health literacy in children	N = 94; *N* = 47 parent-child-dyads *n* = 47 children 7-8y, *n* = 18 (38%) 9-10y, *n* = 18 (38%) 11-12y, *n* = 11 (23%) Science center	Reading comprehension; Numeracy	6 items (provided) (all reading and numeracy)	Up to 3 min	Internal consistency Children α = .71 Parents α = .79	n.a.
15	Naigaga et al., 2015 [54] Uganda English and oga	Maternal Health Literacy Scale (MaHeLi scale) Short form of existing instrument	Subjective measurement/self-report	To use a short form of the validated MaHeLi scale in Uganda	*N* = 384 adolescent pregnant girls 15–19 y. 49% 15 y. 51% 16–19 y. 62% at least level 5 education 38% = < level 5 education Healthcare setting	Appraisal of health information (AHI); Competence and coping (CCS)	12 items (provided); Short version of MaHeLi scale	n.a.	n.a.	n.a.

### Country of origin and setting

Six of the health literacy measurement instruments were developed or applied in the United States [40, 41, 47, 50–52], followed by three from Germany [43, 46, 48] and one each from Greece [42], the United Kingdom (England) [49], Canada [44], China [45], Uganda [54] and Austria [53]. Two articles were published in German [46, 53], and the rest were published in English. While one instrument was used in the general population and not in a specific setting [53], nine studies took place in schools or other educational settings [40–46, 48, 52], four in healthcare settings [47, 49, 51, 54], and one in a mixed setting (educational and healthcare) [50].

### Target or age group

The majority of studies (eleven) provided the exact age of the target group, but this was not specified in the other four studies [41, 44–46]. In these studies, the type of school was specified; three of these studies were performed in secondary schools [41, 44, 46], and one study was performed in both elementary and middle schools [45]. While all instruments have been used in age groups older than 11 years [40–54]; five of these instruments have been used with children 10 years old and younger as well [40, 43, 45, 50, 52], among which only three were specifically designed for children of this age: 9 to 13 years [40, 43], primary school-aged children [45].

### Questionnaire administration mode: Objective and subjective measurement

The analysis showed that seven health literacy tools were based on subjective measurement using self-reporting questionnaires [40, 42, 45, 47, 49, 53, 54]. Another seven articles reported the use of objective measures assessing the actual performance in given tasks [41, 44, 46, 48, 50–52]. One study adopted a mixed-method approach that combined both test methods [43]. All instruments were self-administered measures, except for two that adopted the use of structured interviews to collect the data [50, 53]. Four questionnaires were based on the pen-and-paper mode [46, 51, 52, 54] and another study used a computer-based questionnaire [40]. However, for eight questionnaires the specific administration mode (pen-and-paper or digital) was not provided by the authors [41–45, 47–49]; of these studies, one used two booklets given to the participants indicating that pen-and-paper may have been the mode of administration [44]. Two instruments used a mixture of open-ended and close-ended questions [44, 45], seven used close-ended questions only [40, 43, 47, 49, 52–54], and five did not provide sufficient information on the specific question types [41, 42, 48, 50, 51].

### Participant inclusion in questionnaire development

Only two articles reported the involvement of participants in the development of the questionnaire by conducting qualitative research in order to derive meaning about health literacy from the target population [47, 49]. Massey et al. [47] conducted 12 focus groups with adolescents 13–17 years old and interviewed eight primary care providers who worked with adolescent populations. Chinn et al. [49] conducted expert consultation and focus groups with health and non-health professionals as well as with patients. However, these authors did not report the specific age of the participants. Cognitive pre-testing was conducted by six studies to obtain qualitative feedback by using methods, such as think-aloud techniques or interviews related to question comprehension or feasibility [40, 41, 44, 47–49].

### Psychometric properties

Nearly all the studies reported the psychometric properties of the instruments (see Table 2). The reported reliability differed across measures, with internal consistency ranging from α = 0.42–0.94. The highest reported rates were α = 0.94 [50], α = 0.92 [44], α = 0.90 [53] and α = 0.83 [47]. The weakest internal consistency was reported for the attitude scale α = 0.57 in the GeKoKids questionnaire [43] and for the critical health literacy scale α = 0.42 in the AAHLS questionnaire [49].

Reporting on the convergent validity of most instruments was insufficient. For those provided, these data were positive and moderate (*r* = 0.09–0.93). However, concurrent validities were often not tested or not reported. REALM-teen [50] correlated significantly with WRAT (*r* = 0.83) and SORT-R (*r* = 0.93). TOFHLAd [51] was strongly correlated with WRAT3 (*r* = 0.59) and REALM (*r* = 0.60). HLS-EU-Q-47 moderately correlated with NVS (*r* = 0.14), as did the short version, HLS-EU-Q16 (r = 0.09) [53]. In terms of concurrent validity, the short version was significantly correlated with the long version (*r* = 0.82). Test-retest reliability was reported for one instrument and was demonstrated to be strong (*r* = 0.98) for REALM-Teen [50]. Although the authors of the NVS study claim that the instrument is valid, they further explain that children younger than nine years had difficulties answering the questions [52].

Additionally, Hubbard and Rainey [41] selected items from the HEAP database, which they report to have proven content validity and reliability, as do Massey et al. [47], regarding some of their questionnaire items that were taken from other sources. Schmidt et al. reported the inclusion of some questionnaire items from the German KiGGS survey [55], which have been proven to be valid and reliable [43].

### Health literacy and contextual factors

This review identified five instruments that measured health literacy related contextual factors [42, 43, 47, 49, 53]. These asked for stated satisfaction with healthcare professionals, media sources, or in the context of health learning [42, 47], parent-child communication [43], perceived availability of social resources in form of help and support [49], or perceived difficulties in the social or media interaction [53]. The other instruments focused solely on individual skills or did not provide any information related to contextual factors. However, most studies focused on three main context arenas: healthcare, prevention, and health promotion, including health education (see Table 2).

### Underlying health literacy understandings

Each instrument used a different, study-specific understanding of health literacy, and two instruments were underpinned with a health literacy definition that was specifically developed for the instrument [40, 53]. In the other 13 studies [41–52, 54], the researchers referred to different existing definitions but did not make clear whether or not these definitions were underlying their instruments, and of these, six studies [42, 43, 46–49] referred to the definition provided by Nutbeam [8, 56]. A further five articles [42, 47, 48, 51, 52] quoted the Healthy People 2010 definition [57], the definition of Ratzan and Parker [58] or that of Parker and Ratzan [9], and the definition by Zarcadoolas and Pleasant [59]. One study [54] did not refer to a specific definition. Instead, the authors emphasised two different models as important for their tool, the HLS-EU model [1] and the Health Belief Model (HBM) [60]. Finally, one study [46] referenced the definition developed by Mancuso [61].

The analysis revealed three different conceptual approaches that underpin the instruments: health literacy was based on (i) a functional literacy model in three studies [50–52], (ii) school health education standards from different countries in five studies [40–42, 45, 46], and (iii) broader multidimensional models in seven studies [43, 44, 47–49, 53, 54]. In one of the studies the researchers claimed that their instrument measured functional health literacy by assessing knowledge, whilst the knowledge questions were based on school health education curriculum [46]. Although knowledge is highlighted as a main component of functional health literacy [8], this instrument was nevertheless instead classified under the second category rather the ‘functional literacy’ category.

### Scope of measured components

**Fig. 2:**
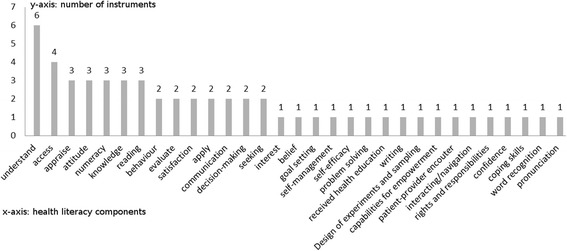
Health literacy components used in the instruments

To ascertain the scope of the measurement approach, a content analysis of the measured components (sometimes referred to as “dimensions”) was performed that could identify a wide array of skills, competencies, abilities, or certain actions covered by the instruments. To closely relate to the original published study and thereby avoid any false interpretation, we decided to label a specific component as if the authors of the original article had named it explicitly. For example, in Brown et al. [40], the authors explicitly state that their instrument measures understanding, accessing, and applying health information as well as beliefs, attitudes, and interests towards health. Based on this, six components could be identified: understand, access, apply, belief, attitude, and interest. In Chisolm and Buchanan [51], the authors explain that their instrument intends to measure reading ability and numeracy skills. Therefore, we extracted two components, reading and numeracy. We applied this coding scheme to all identified articles. In total, the 15 instruments comprised 31 different components (see Table 3 and Fig. 2). The ability to understand health information was identified in six studies [40–42, 44, 48, 53] and was the most prominent approach to measure health literacy, followed by four studies that asked about the ability to access health information [40, 41, 49, 53]. The health literacy components appraise, attitude, communicate, knowledge, reading, and numeracy were found in each of the three instruments. Many different components, such as problem-solving, self-management, coping, and self-efficacy, were each assessed in only one instrument.

**Table 3:** Scope of measured components

Component	Study no. (for study numbers see Table 2)														
	1	2	3	4	5	6	7	8	9	10	11	12	13	14	15
Understand		X	X		X	X		X					X		
Access		X	X							X			X		
Apply		X											X		
Interest		X													
Belief		X													
Attitude		X					X		X						
Reading				X						X				X	
Communication			X				X								X
Decision-making			X												
Goal-setting			X												
Self-management			X												
Numeracy				X	X									X	
Seeking					X							X			
Design of experiments & sample					X										
Knowledge							X		X		X				
Behaviour (health practice)							X		X						
Self-efficacy							X								
Capabilities for empowerment							X								
Satisfaction (i.e. asking, requesting, etc.)						X									
Received health education						X				X					
Evaluate								X							
Writing										X					
Appraisal										X			X		X
Patient-provider encounter										X		X			
Interaction / Navigation												X			
Rights and responsibilities												X			
Confidence												X			
Coping skills															X
Problem solving															X
Word recognition	X														
Pronunciation	X

## Discussion

The main objective of this review was to systematically identify, retrieve, analyse, and assess available generic health literacy measurement tools for children and adolescents ≤18 years old. Despite the gap in health literacy research on children and adolescents, our search found 15 available generic measurement tools published between 1990 and 2015. This is the second known systematic review on health literacy measurement tools for children and adolescents. The previous one was conducted by Ormshaw et al. [38] and considered tools published until 2011. By encompassing domain-specific health literacy measures as well, their review had a somewhat different focus than this present review. Ormshaw et al. identified 16 tools for children and/or adolescents, including tools for measuring mental health literacy, media health literacy, or oral/dental health literacy. It should also be noted that their review included studies using the same instrument, for example, TOFHLAd and questions based on the National Health Education Standards (NHES, USA), whereas the present review includes an instrument only once. Therefore, the present study only has six instruments in common with that review [40, 41, 43, 44, 48, 51].

### Country of origin and setting

While seven health literacy instruments (nearly 50%) have been used in North America [40, 41, 44, 47, 50–52], all but one [44] in the USA, six instruments have been developed and used in Europe [42, 43, 46, 48, 49, 53]. However, health literacy measurement tools for children and adolescents remain a marginalised area of research in Asia [45] and Africa [54], with only one instrument found for each region. The authors of this review are aware of instruments used in Asia that had yet not been published in English while preparing this article [62–64]. However, our search has not identified any generic health literacy instruments for South America or Australia. Given that Australia is one of the pioneering areas of health literacy research and action in childhood and adolescence, primarily in the context education [28, 65], this result is especially surprising. In relation to Africa, while the short form of the MaHeLi scale was adapted to the settings in Uganda, it was originally a European-based instrument [54]. Health literacy research in Africa, a continent with approximately 60 countries with more than one billion inhabitants and serious health threats to children and adolescents [66], has only recently begun and thereby is calling for an ‘African health literacy scheme’ and proportionate solutions [67]. Given the social, cultural, economic, and political differences between regions, it is especially important that research in these regions engages in health literacy with children and adolescents to facilitate better health promotion despite the challenges that these populations face. Development of more culturally sensitive concepts and measurement tools in these regions would also support this approach. In turn, and due to migration to Western countries from within these regions, health promotion and prevention in migrant and refugee child/adolescent populations in Europe and North America in particular could benefit by such approaches when adapting these tools and concepts.

Regarding the settings in which health literacy was measured, it seems that schools and other educational settings are main loci of interest for measuring the health literacy of children and adolescents. This review found that many health literacy tools are based on school health topics or curricula [40–46, 48]. When researchers aim to derive an understanding of health literacy for children and adolescents, an existing health literacy-related school curriculum could provide the necessary information and would also ensure comparable instruments and models. Shaping of school health topics to match health literacy content, therefore, would foster further development and the use of comparable instruments, at least within countries, which would allow for consistent monitoring and evaluation of health literacy in these settings.

### Target or age group

The findings of this review suggest that the age groups from 11 to 18 years are well reflected by existing health literacy tools (see Table 2). This review was able to identify a total of 15 instruments that were used with children within those age groups [40–54]. However, only five of these tools had been used to measure health literacy of children younger than 11 years old [40, 43, 45, 50, 52]. One instrument was used with children aged 7 to 12 years old within a parent-child study [52]. In this study, the authors reported difficulties for children younger than 9 years old when administering the questionnaire [52]. This finding is supported by another study using this instrument in that age group [68]. One instrument was used in a study specifically conducted in the primary school setting in China without providing the exact ages of the children who participated [45]. However, we assume that the children were most likely between 6 and 10 years old, which is the common age of primary school-aged children in China. The same study also used their instrument with secondary school children, but they did not provide any information on differences in using this tool between age groups. Two instruments were used with secondary school children aged 9 to 13 years [40, 43]. Although it is most common that children 9 and 10 years old usually attend primary schools rather than secondary schools, in those two studies, the children were nevertheless reported to be students at secondary schools. Together with the Chinese instrument [45], these two [40, 43] were also the only instruments that were specifically developed for children younger than 11 years old. The other two instruments were adaptations of the REALM [50] and NVS [52] studies. With only one instrument specifically developed for the use in primary school-aged children [45], our findings emphasise that there might be only one health literacy instrument available for primary school children. Unfortunately, the authors do not provide adequate information that might facilitate a better understanding of their instrument. In conclusion, there is a lack of adequate information on health literacy instruments for primary school children and no instruments available to assess the health literacy of children younger than seven except the Chinese tool [45].

Health literacy is important for young children, but the conceptualisation and measurement of health literacy are different from the approach taken with adults. Existing health literacy concepts specific to adults address how people understand, evaluate, and use health information, which may not be appropriate for children of primary school age who are not making all the health-related decisions that affect them. Not only do such approaches demand more complex skills to process health information, but an individual also needs more experience and autonomy to be able to judge the quality of information or to act based on given, sometimes complex, information. Primary school-aged children are still developing their formative skill sets that are required to process health information, and they will have acquired far less health-related knowledge and experience and have less autonomy than adults or older children [14, 17, 18, 20, 22, 26–28]. Therefore, health literacy concepts and their assessment in this age group have to be less complex and should focus on more basic aspects of health promotion and education, such as healthy eating, physical activity, or knowledge about their bodies and the environment. Arguably in healthcare contexts, the health literacy of the parents of young children is more likely to be important and have greater impact than is that of the young children themselves, as the parents are those who communicate with professionals and make the influential health-related decisions. These assumptions are also supported by two studies that reported on cognitive difficulties experienced by younger children during the administration of health literacy instruments [52, 68].

### Questionnaire administration mode: Objective and subjective measurement

Half of the identified tools were subjective measurement tools and asked the participants for an assessment of their self-perceived health skills and half were objective measurement tools and assessed the actual capabilities by performance testing. Only one tool combined both assessment methods [43]. Although self-reported health literacy measurement has been used in many studies and instruments [4, 32], there is much criticism concerning self-reporting as a valid measure of health literacy [33, 44, 49, 69–72]. It is argued that self-reporting measures are oversimplifying the given complexity of health literacy [49], or, in some cases, assess self-efficacy rather than health literacy [33]. Furthermore, a discrepancy between self-reported health literacy and the actual performance related to health literacy has been highlighted indicating concerns with self-reporting measures in terms of accuracy [33, 44, 49]. Other research suggests that self-reporting methods are just as valid as objective methods [73]. Ethical concerns related to objective measures, however, have highlighted the potential discomfort that participants with low health literacy skills may feel if they are ashamed or embarrassed by their abilities [73, 74]. For future health literacy research with children and adolescents, adopting a combination of both methods might prove a valuable way in order to generate more detailed and profound data. It would also allow for the comparison of differences in health literacy as measured with these two different methods. Applying different research methods may also generate richer results and, therefore, would support the development of better and more problem-centred interventions to address specific health literacy weaknesses.

### Participant inclusion in questionnaire development

Including participants in the development of a health literacy questionnaire has proven to be a sound method to improve the quality of the measurement tool [75]. Regarding the development of new and innovative health literacy tools, it is recommended always to include the target population in the development process as early as possible [76]. However, analysis of this review showed that children are mostly excluded from the development of the instrument and/or the conceptual construction that underpins the questionnaire. With only two reporting qualitative research with adolescents and/or adults during the development process of the instrument [47, 49], child and adolescent involvement was poor. In none of the studies were children younger than 13 years old actively involved, except for completing the questionnaires. Accordingly, this review found adult experts setting out to define a concept for children and young people without consulting them to determine their understanding of health literacy and assuming the health-related skills and knowledge and health behaviours and practices that may be important for them in their everyday lives. This conflicts with theories and findings from childhood studies that consider children and adolescents to be active citizens, social agents, and co-constructors of their social worlds [77, 78]. Researchers from this field also highlight that children should be understood as a social minority living in a “childhood” with unequal power relations, that they suffer from an uneven distribution of rights, and that one major weakness in research and practice is that childhood is mainly constructed by adults [28, 77, 78]. Although two studies valued contributions from adolescents as useful sources of qualitative information, there is considerable room for improving child/adolescent participation in the health literacy research process, especially as there are yet no studies including young children in the development of instruments. Therefore, little is known about what health literacy means to children, what knowledge and skills are important to them in order to promote their health, or how, where, when, with whom, and why (or why not) they are interested in developing skills in order to promote their own health. To learn more from children and to learn collaboratively with them, their active involvement and participation in the social and cultural construction of health literacy and its measurement is a specific challenge that would be highly beneficial to overcome for research with children. In not doing so, an important voice, the children’s perception of health and health literacy, is not recognised and given the consideration that it deserves in the field of health literacy research. Although, few studies have performed qualitative health literacy research with children [29, 62], in recent years, especially in health-related disciplines (for example, mental and dental health), qualitative research with children has increased [79–82]. Researchers argue that the benefits of children’s involvement by using qualitative research methods are obvious and include enhancing child empowerment, producing better knowledge and understanding of children’s views and priorities, and developing better tools and practice measures for more effective action [79, 81]. Additionally, as such, it could be argued that applying qualitative methods in health literacy research with children would have similar effects and thus produce more precise findings and unravel children’s own perspectives and knowledge related to health. Such findings could be used to derive a child-centred understanding of health literacy and elucidate the underlying tenets of children’s health literacy. Furthermore, children’s participation could also support the development of more child-adjusted measurement tools and facilitate the development of tailored health literacy interventions that better match the needs and demands of this population.

### Psychometric properties

The results concerning the validation processes are diverse. Only a few studies provide validation data on the instrument [44, 48–51, 53], and most instruments have weak or only moderate validity. The focus of reporting in these studies is on statistical population data, results, and sample characteristics, while reporting on methodological data and psychometric properties is scarce and not well described. These results confirm findings from other systematic reviews with different foci [31, 32, 37]. Only one article made test-retest-reliability data available [50]. To support better clarity on the characteristics of tools, the authors should provide more detailed information on the development and validation process, psychometric properties, and assessment characteristics.

### Health literacy and contextual factors

There has been much debate in the conceptual and methodological discussion regarding adult populations as to whether health literacy is only associated with individual abilities, or if beyond individual abilities, context also might have an essential impact on the health literacy of individuals [1, 9, 10, 16, 21, 33, 58, 59, 70, 71, 83–85]. Similarly, the influence of context is also discussed within the scientific literature related to children’s health literacy [14, 20, 22–28]. It seems that the scientific community generally agrees that context is an important dimension that should be considered appropriately and that health literacy itself is a relational concept that is influenced by more than only individual abilities. In general, when the relatedness of health literacy is being discussed, the context dimension is mainly meant to capture the complexities and demands of the health system and the health literacy of health professionals, particularly in relation to the health literacy of individuals that they interact with in certain health literacy related situations [9, 16, 58, 85]. This also includes information materials being tailored and user-friendly and communication being based on understandable and simple language [85]. In this context, the identified measurement tools, however, did not provide much information because most of the tools focused on measuring individual abilities rather than contextual factors with impact on health literacy. However, when the context dimension is extended to also address contextual factors beyond health system complexities, there are some findings that can be reported. Overall, the analysis could identify five instruments that included questionnaire items addressing contextual factors or situational determinants that might influence health literacy [42, 43, 47, 49, 53]. In relation to health literacy, one instrument assessed the stated satisfaction of adolescents in the context of health learning in schools [42]. Another questionnaire asked adolescents about the confidence that they feel when they try to access health information from personal and media sources [47]. Two instruments included broader context questions in relation to self-perceived difficulties with respect to health literacy and thereby asked about the trustworthiness and meaning of health information and sources, the possible impact of the social and political environment on health, and the interaction with health professionals [49, 53]. Finally, one instrument included questions related to children’s communication habits with their social environment (friends/parents) [43]. Apart from collecting data on individual abilities, asking such questions related to emotions, attitudes, opinions, and interests within the context of specific health literacy situations seems to be a promising approach that could support the derivation of a broader understanding of the interplay between individuals and their health-related environment. In addition, it allows for encountering valuable insights from individuals and how they perceive the influence of context on their health literacy and health practices.

Context can also be understood as the setting in which individuals use their health literacy or interact with others, such as in healthcare and medicine, disease prevention, health promotion, everyday life, and educational settings. In this context, our review has found several health literacy instruments that are related to different contexts and their inherent characteristics. While the health literacy instruments used within the educational setting had a dominant focus on health promotion resources and prevention [40–46], studies conducted in hospitals or medical centres addressed various aspects of the healthcare environment by asking questions, i.e., on treatment, self-management, and interaction with health professionals [47, 49–52, 54]. Two instruments were found that addressed mixed contexts, including healthcare, prevention, and health promotion and education [48, 53].

However, to provide meaningful data on the impact of context on individual health literacy, future health literacy measurement tools for children and adolescents should include more questions related to contextual factors. In addition, tailored questionnaires could also be administered simultaneously in dependent populations within the same study, for example, involving teachers and students, patients and health professionals, or parents and children, including questions on the various inherent intricacies of complex contexts as discussed within the conceptual health literacy literature. This would ensure the capture of both sides of the health literacy concept, the individual and its related context.

### Underlying health literacy understandings

Health literacy measurement tools for both adults and children/adolescents have been criticised for different reasons [33, 38, 86]. First and foremost, the lack of a consistent general understanding of health literacy is highlighted as a major weakness within the field [1, 33, 76, 85] that affects the development of comparable methods to accurately measure the concept [33, 85]. This review supports this finding, as each of the identified instruments introduced an individual and/or study-related understanding of health literacy. This makes it difficult to compare the instruments or the results generated by these instruments. Even instruments that apply similar conceptual approaches vary considerably in how they transfer the underlying concepts into questionnaire items at the operational level. Three instruments, for example, measure functional literacy [50–52], which can be described as a unidimensional approach to health literacy and sometimes is referred to as medical health literacy [1, 83, 84, 86]. Even though REALM-teen [50], TOFHLAd [51], and NVS [52] are tools that measure functional literacy, they are however testing different components of the literacy construct, such as reading, comprehension, and numeracy. Another tool addressing functional ‘health literacy’ is the “health quiz” measurement tool [46]. However, it is based on the functional concept of health literacy as proposed by Nutbeam [8], measuring knowledge rather than literacy, as in the previously mentioned tools [50–52].

Compared to the narrow ‘functional literacy’ approach to health literacy, all other instruments (*n* = 12) are informed by a multidimensional, multifactorial, or multifaceted and thus broad understanding of health literacy. Still, each understanding is more or less unique, and therefore, comparisons across these health literacy assessment instruments and associated study results are difficult to undertake. Additionally, as the instruments do not cover all their proposed dimensions and components at the operational level, there seem to be some uncertainties regarding how to transform all aspects of the theoretical model into a testable concept within a measurement tool. It is also not clear why a certain model or definition is provided at the conceptual level when at the operational level, something else is indeed being measured. However, when school health education (or other educational contents and contexts) is the foundation of the health literacy framework, it seems that there is more precise clarification as to why, how, and for what purpose a model was designed and operationalised [40, 42–45]. If an instrument has been based on a clear conceptual model, it is much more feasible to clearly understand what is assessed by the construct [43, 47–49, 53, 54]. However, in some studies, the operational definition covers only select aspects of the conceptual definition [40, 41, 43, 53, 54].

### Scope of measured components

When researchers measure health literacy, there is a wide array of components at the operational level that can be assessed by instruments [4, 38]. This review was able to identify a total of 31 different components that were addressed by 15 different instruments (see Table 3). As these studies each provide a different understanding of health literacy, including the chosen components to reflect upon, neither the conceptual/operational definitions nor the instruments were found to be comparable to one another, although some instruments apply and assess similar components. Moreover, not all authors provide their questionnaire items or make them accessible elsewhere, which makes understanding the tools difficult and prevents an accurate evaluation of exactly how each component was measured or indeed if it was measured at all. Thus, it is not possible to assess how far the predefined concept is represented in the questionnaire items or scales. Accordingly, we argue here that authors should make it quite clear which aspects of the model they intend to measure and which they actually do measure, and explain why they measure in that way. Of course, providing the questionnaire items could additionally support a better understanding of the instrument and the operationalisation of the underlying concept, if applicable. Despite the importance given to the social and cultural dimensions of health literacy [1, 8, 14, 16, 21, 26, 27, 56, 59], most instruments fail to address measurable components related to cultural competencies or social skills making it difficult to evaluate their actual impact on individual’s health literacy. Further components that are considered important to health literacy, such as empowerment, attitude, and self-efficacy [8], can be found in few instruments [40, 43, 45, 47].

### Limitations

This review has several limitations. Not all identified studies provided the questionnaire items which makes it difficult to undertake an accurate evaluation of the instrument without knowing how the construct was operationalised. This becomes even more critical if the components are only loosely described. In some cases, in which studies did not provide the questionnaire, the authors could be asked to provide the instruments to undertake further analysis with more detail. Unfortunately, several promising studies on health literacy measurement tools for children and adolescents, were not available in German or English [62–64]. In the systematic review of Ormshaw et al. [38], further domain-specific and generic health literacy measurement tools for children and adolescents are reported. Having also analysed these studies during our review process, the authors of the original studies cited by Ormshaw et al. [38] did not provide necessary information to match the inclusion criteria of this review. Either some of the described instruments were used in studies we had already included, such as adaptations of TOFHLA, REALM, and NVS, or the articles did not describe the instruments in the necessary detail. Thus, these instruments were not included in this present review. However, these instruments may provide useful information for the development of new health literacy tools for children and adolescents. Furthermore, as health literacy is seen by some as a repackaging of the already existing concepts of health promotion and education, such as empowerment, self-efficacy, life skills, and the community approaches [87, 88], it may be useful to consider the methodology by which these concepts are being measured. For example, life skills measures could provide useful information for health literacy as well. This impression is supported by Sørensen and Brand [89], who reflected on the different translations and contexts of health literacy in which life skills or health-related competencies are used sometimes as synonyms for health literacy.

Regarding general limitations in the context of the health literacy measurement tools that were investigated during this study, the measurement of health literacy in children and adolescents is particularly difficult for two reasons. Firstly, there is no commonly accepted health literacy model or definition. In fact, there are approximately 250 different definitions or models available for adults [90], while there is a significantly smaller number available for children and adolescents [91]. Various constructions co-exist but are indeed fundamentally different, and they are apparently associated with uncertainties with respect to their measurement. The understanding that informs an assessment tool defines what kind of health literacy interpretation is measured. It is no surprise that across studies and tools dedicated to the assessment of health literacy, health literacy itself can be a different construct in each case. To progress in the field of measurement tools, consensus is essential in the conceptual field. Secondly, and in addition to conceptual concerns, another important issue is related to the cognitive and social development of children, which should be seriously considered when addressing the measurement of health literacy. Developing adequate health literacy levels requires certain prerequisites, such as skills; knowledge; level of experience with regard to health; autonomy and independence; and acquired social skills. Considering current conceptualisations of health literacy and how they are being measured, it is questionable whether young children will have acquired the competencies required to undertake the complex processing of health information as demanded by most of the existing health literacy models. As the achieved skill sets of children vary enormously across age groups and diverse backgrounds, there might be a need for age-appropriate and developmental-stage-adjusted concepts and their operationalisation considering the actual (social and cognitive) capabilities of children at different ages during childhood and adolescence.

## Conclusion

This article sought to systematically review the field of children’s and adolescents’ generic health literacy measurement instruments and provides a detailed analysis of these instruments. Health literacy research with younger age groups is a growing field. However, methodological approaches require further improvement. In terms of conceptual and operational definitions, it seems that instrument development first needs a clear conceptual understanding of health literacy that should be transferable to an operational definition that covers all aspects of that understanding [33]. This is supported by findings presented in this review, and similar observations on theoretical definitions not matching the operational definition have been made [37, 47, 85, 92]. Furthermore, given the lack of a specific and explicit health literacy definition and/or if the understanding is vaguely based on different definitions and models, it is far more difficult to understand how the conceptual model has been operationalised. To date, there is scarce data for constructing an effective blueprint for health literacy measurement instruments. Furthermore, there is currently a specific gap regarding health literacy measurement instruments aimed at primary school and early childhood-aged children. From a methodological perspective, when health literacy instrument studies are designed, especially for children, they should consider a mixed-method approach combining both subjective and objective measurement approaches. This would allow for the comparison of results and would secure the validity and reliability of the instruments. Although there are currently no findings from general health literacy research with children that specifically articulate the involvement of children in the development of measurement tools, research with children conducted in other disciplines shows the benefits of children’s involvement. Children’s meaningful involvement in health literacy research could be as beneficial as it is currently in dental health and mental health research. It could, for example, enlighten researchers in terms of a better understanding of children’s views, interests, perceptions, feelings, interactions, and worlds, which then could be used to develop models and measurement tools that are better suited to children. Furthermore, recent studies highlight the importance of meeting the specific health literacy needs of children and adolescents [5, 20, 28]. This includes development of materials and information that are suited to younger age groups and provided in ways that engage and empower them or improve uptake [5, 26, 27]. This may well foster an improvement in the development of health literacy throughout life, beginning in early childhood.

Currently, the valid measurement and assessment of child and adolescent heath literacy are gaining importance in terms of monitoring and evaluating the effects of health literacy promotion in children and adolescents. In accordance with this, many scholars have recently called for advancing health literacy measurement [33, 85, 86, 92, 93], including child-specific instruments [26, 38]. With this work, the authors hope to stimulate further scientific research and action, especially concerning health literacy measurement development as well as intervention and policy development. The results of this review will be of value and considerable interest to researchers and practitioners interested in health literacy measurement as it explains which instruments already exist and how they were developed, applied, tested, and validated.

## Additional files

Additional file 1: PRISMA study protocol. (PDF 384 kb)

Additional file 2: Search methodology. (PDF 201 kb)
