# Supplementary material for: Generic health literacy measurement instruments for children and adolescents: a systematic review of the literature
Source: BMC Public Health. 2018 Jan 22;18:166. doi: 10.1186/s12889-018-5054-0 (PMC5778701; doi:10.1186/s12889-018-5054-0)
Supplement: Supplementary file 2 — Search methodology. (PDF 201 kb) [file 12889_2018_5054_MOESM2_ESM.pdf]

1. PubMed

Three search blocks, with study population restricted to children and adolescents and youth

Search results: n=291

Search algorithm:

((health literacy[Title/Abstract]) AND ((child*[Title/Abstract]) OR (adolescen*[Title/Abstract]) OR (youth[Title/Abstract])) AND ((measur*[Title/Abstract]) OR (test*[Title/Abstract]) OR (tool*[Title/Abstract]) OR (instrument*[Title/Abstract]) OR (questionnaire*[Title/Abstract]) OR (assessment*[Title/Abstract]) OR (screen*[Title/Abstract]) OR (survey*[Title/Abstract]) OR (psychometric*[Title/Abstract]) OR (review*[Title/Abstract])))

American Psychological Association APA PsycNet

Including following databases:

PsycINFO; PsycARTICLES; PsycBooks; PsycEXTRA; PsycCRITIQUES; PsycTESTS

Search results: n=357

Search algorithm: Results were narrowed by following categories:

Any Field: Health Literacy AND Index Term: Health Literacy AND Age Group: Infancy (2-23 mo)

(n=16)

Any Field: Health Literacy AND Index Term: Health Literacy AND Age Group: Preschool Age (2-5 yrs)

(n=29)

Any Field: Health Literacy AND Index Term: Health Literacy AND Age Group: School Age (6-12 yrs)

(n-56)

Any Field: Health Literacy AND Index Term: Health Literacy AND Age Group: Childhood (Birth-12 yrs)

(n=95)

Any Field: Health Literacy AND Index Term: Health Literacy AND Age Group: Adolescence (13-17 yrs)

(n=161)

CINAHL via EBSCOhost

Search results: n=201

Search algorithm: Boolean phrase used as follows:

TI Health Literacy

Limiters: “all Child”

---

4. ERIC via EBSCOhost

Search results: n=226

TI "health literacy" OR AB "health literacy"

---

5. FIS

Search results: n=57

Search algorithm: Boolean phrase used as follows

Gesundheitskompetenz

(((Titel: GESUNDHEITSKOMPETENZ) und (Freitext: KIND*)) oder (Titel: GESUNDHEITSKOMPETENZ)) und (Freitext: JUGEND*)

Search results: n= 5

(((Titel: GESUNDHEITSKOMPETENZ) und (Freitext: SCHUEL*)) oder (Titel: GESUNDHEITSKOMPETENZ)) und (Freitext: SCHUL*)

Search results: n=1

GESUNDHEITSBILDUNG

(((Titel: GESUNDHEITSBILDUNG) und (Freitext: SCHUEL*)) oder (Titel: GESUNDHEITSBILDUNG)) und (Freitext: SCHUL*)

Search results: n=44

(((Titel: GESUNDHEITSBILDUNG) und (Freitext: KIND*)) oder (Titel: GESUNDHEITSBILDUNG)) und (Freitext: JUGEND*)

Search results: n=7

The search process identified **N=1132** publications matching the search criteria (Pubmed n=291; CINHAL n=201, PsycNet n=357, ERIC n=226, FIS n=57). The hand search lead to the identification of additional n=2 articles (Fig. 1: PRISMA flow diagram). After removing duplicates, n=764 articles remained, of which a further n=437 articles were excluded after screening the titles and abstracts. A total of n=327 articles underwent full-text analysis. Finally, all articles not matching the inclusion criteria (n=312) were excluded from qualitative synthesis yielding n=15 articles reporting 15 different questionnaires.
